# Acute Angle Closure in an 18-Year-Old Due to Plateau Iris

**DOI:** 10.7759/cureus.60608

**Published:** 2024-05-19

**Authors:** Sohum Sheth, Mollie Lagrew, Charles Richard Blake

**Affiliations:** 1 Ophthalmology, University of Florida College of Medicine, Gainesville, USA

**Keywords:** plateau iris syndrome, argon laser iridoplasty, child, plateau iris, angle-closure

## Abstract

In this case report, we describe a rare case of acute angle closure in an 18-year-old African-American female, attributed to plateau iris. The patient had no significant medical or ocular history and presented with high right-eye pressure, headache, and blurred vision. Ocular examination revealed findings consistent with acute angle closure, with gonioscopy confirming superior iris insertion anterior to Schwalbe’s line and a "double hump" sign. Ultrasound biomicroscopy confirmed plateau iris. Treatment involved pharmacological management and bilateral peripheral laser iridoplasty. This case underscores the importance of considering plateau iris syndrome in the differential diagnosis of acute angle closure, even in younger patients, and highlights the role of early diagnosis and appropriate intervention in preventing vision loss.

## Introduction

Plateau iris is characterized by a normal anterior chamber depth with a larger or more anteriorly positioned ciliary body, which mechanically angulates the peripheral iris forward and renders a flat, planar iris more centrally [1]. The anterior displacement of the ciliary body may lead to occlusion of the angle and obstruct outflow through the trabecular meshwork [1]. Plateau iris configuration refers to the condition in which plateau iris and an appositional or narrow angle are confirmed by gonioscopy.

In adults aged 30-50 years, plateau iris is one of the possible causes of angle closure and can lead to chronic angle closure glaucoma [1]. In patients with narrow angle or primary angle closure glaucoma who underwent ultrasound biomicroscopy, the prevalence of plateau iris was 9.6% [2]. Typically, plateau iris affects middle-aged women, with a predilection for Caucasians and those with a family history of angle closure [3]. In children and teenagers, the presentation of acute angle closure is uncommon, especially in patients without a history of retinopathy of prematurity or connective tissue disease (i.e. Weill-Marchesani) [1,4]. Here we report a rare case of plateau iris configuration in an 18-year-old.

## Case presentation

An 18-year-old African-American female with no significant medical or ophthalmic history presented to an urgent walk-in clinic with a one-week history of “on and off” right-sided headache and blurred vision. By the time of the initial exam, the headache was constant for two days and she reported nausea, vomiting, photophobia, and right-eye pressure.

Intraocular pressures were 27 mmHg in the right eye and 12 mmHg in the left eye. Slit lamp exam revealed no corneal abrasions without obvious anterior chamber cells or flare, after which the patient was transferred to the main hospital for ophthalmology evaluation.

On arrival at our emergency department, the patient was found to be in acute pupillary block angle closure in her right eye. Intraocular pressure was 36 mmHg in the right eye. Anterior chambers were deep centrally and narrow peripherally in both eyes, with the right-eye slit lamp exam demonstrating pigment clumping on corneal endothelium without anterior chamber cells or hypopyon. Fundoscopic exam showed a healthy nerve with good rim tissue. Gonioscopy of the right eye revealed superior iris insertion anterior to Schwalbe’s line at 35 degrees of angular approach with the peripheral iris bowed anteriorly. Visualization of the inferior angle showed some trabecular meshwork, which was heavily pigmented. Laser peripheral iridoplasty was recommended but deferred by the patient and family, instead opting for medical therapy until consultation with a trusted ophthalmologist known to the family. Latanoprost qHS, brimonidine TID, dorzolamide TID, timolol BID, diamox 250 mg BID per os (PO), and pilocarpine 2% QID were initiated at discharge and the patient was seen the same day in the clinic.

**Figure 1:**
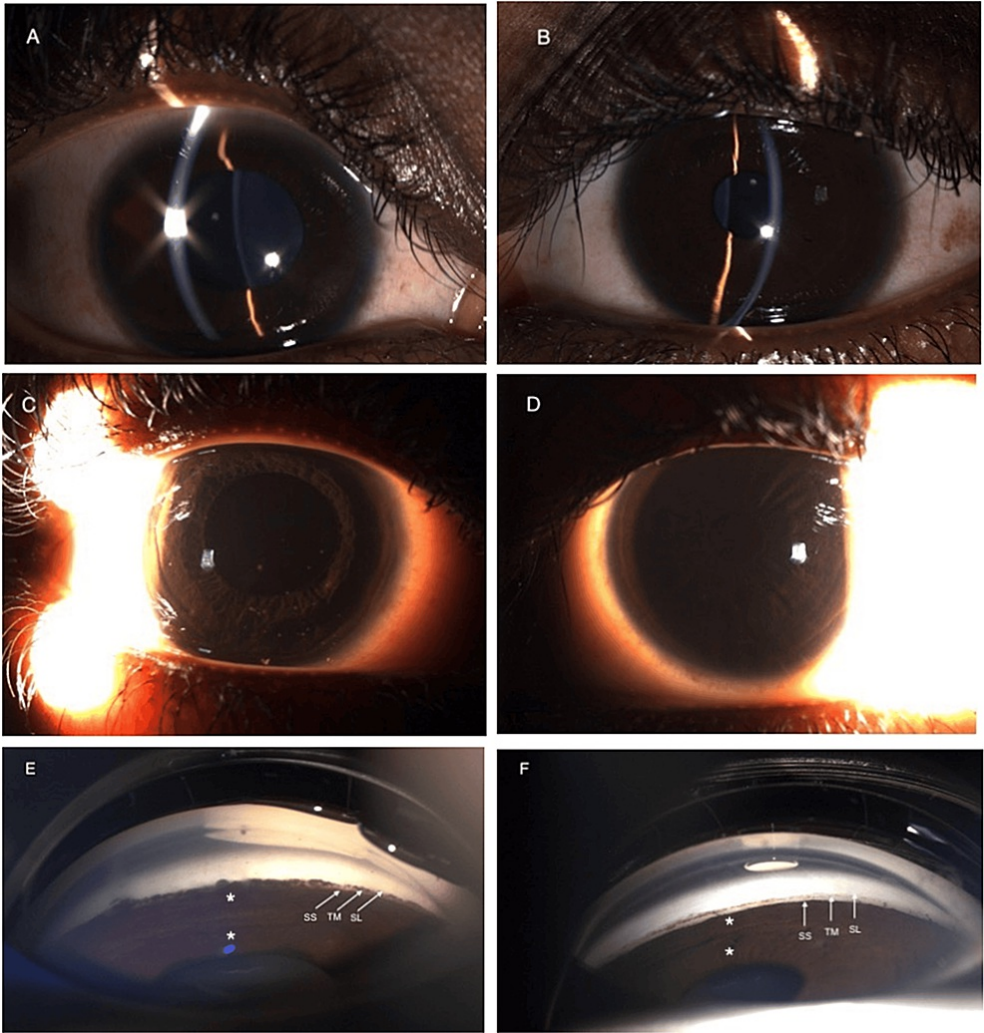
A,B: Plateau iris configuration on slit lamp examination bilaterally, with anisocoria. C,D: Sclerotic scatter illuminates corneal opacities in the right eye. E,F: Indentation gonioscopy showing the double hump (*) characteristic of plateau iris, along with the scleral spur (SS), trabecular meshwork (TM), and Schwable’s line (SL). Images on the left are OD (right eye) and images on the right are OS (left eye).

**Figure 2:**
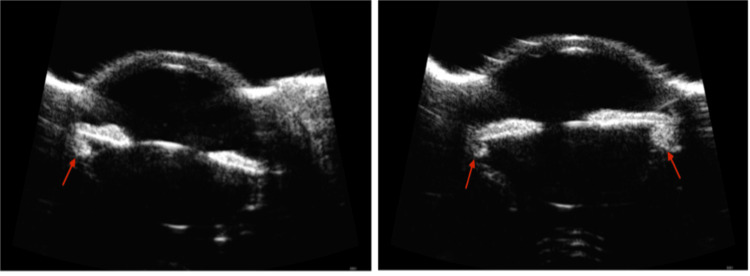
Ultrasound biomicroscopy imaging demonstrating plateau iris configuration bilaterally (left image: OD [right eye]; right image: OS [left eye]). In both eyes, a flat iris plane is seen along with anteriorly positioned ciliary bodies (red arrows).

In the outpatient clinic, the patient was reevaluated with slit lamp examination and gonioscopy, which demonstrated a plateau iris configuration (Figure 1). Ultrasound biomicroscopy was performed to assess ciliary body anatomy, revealing the anteriorly positioned ciliary body (Figure 2).

Peripheral laser iridoplasty in the right eye was performed after the patient was treated with pressure-lowering drops, including pilocarpine. Prednisolone acetate was started on the first patient visit to quiet the eye before treating with iridoplasty. Iridioplasty was performed utilizing Pascal laser (double-frequency YAG (yttrium aluminum garnet) laser) with a spot size of 400.0 μm and laser power of 300 mJ, with a total of 48 spots in a 360-degree fashion. Peripheral laser iridoplasty for the left eye was performed four weeks later to prevent angle closure (initially she was treated with pilocarpine 2% QID).

## Discussion

In this report, we present a rare case of acute angle closure in an 18-year-old female due to plateau iris. In children or teenagers, the differential for acute angle closure can be challenging and includes iridociliary cysts, uveitic glaucoma, nanophthalmos, iris tumors, anterior segment dysgenesis, microspherophakia, and plateau iris. Reports of plateau iris are typically not common and, if diagnosed in adults, the mean age of presentation is 40 years [3]. For some clinicians, plateau iris is a diagnosis of exclusion in cases where peripheral iridotomy or cataract removal do not help [3]. While plateau iris is rare in younger patients with limited reference in the literature in this population [5,6], it should be considered in the examination and in the differential diagnosis of young patients to mitigate the risk of bilateral glaucoma.

Plateau iris is often missed when using Van Herick slit lamp technique to assess angle closure. Unlike typical angle closure from pupillary block, anterior chamber depth is normal on a slit lamp examination. On gonioscopy, a flat iris plane with steep downward angulation is noted. Classically, a “double hump” sign is seen after indentation. The peripheral “hump” is created from the iris anteriorly displaced by the ciliary body and the central “hump” due to the iris over the anterior lens surface. While ancillary imaging, such as ultrasound biomicroscopy (UBM), is useful to inform anterior segment anatomy, gonioscopy enables a dynamic examination, which is helpful for diagnosis. Anterior segment optical coherence tomography (AS-OCT) has poorer diagnostic performance in the detection of plateau iris configuration than UBM and should not replace UBM in evaluation [7]. This report highlights the value of performing UBM to arrive at a proper diagnosis, especially in rarer cases involving young patients. 

Peripheral laser iridotomy is the typically first-line surgical intervention [8]. Similar to the current practice of peripheral iridotomy in primary angle closure patients, peripheral laser iridotomy of the other eye to prevent angle closure could be considered. Anterior chamber anatomy is not modified with iridotomy, so plateau iris patients following peripheral laser iridotomy are still at risk for further secondary angle closure and development of additional peripheral anterior synechiae. One report [9] noted a plateau iris syndrome prevalence of 54% despite initial iridotomy or iridectomy. As iridotomy does not modify the underlying disease mechanism, these patients may be better treated with iridoplasty, especially in cases where pupillary block is not acutely present [10]. Peripheral laser iridoplasty opens the angle in plateau iris by not only causing iris stroma contraction but also thinning the iris tissue at the angle [10,11]. Pilocarpine administration can induce miosis and minimize the chance of iris entrapment of the angle. Ritch and colleagues [12] evaluated the long-term success of iridoplasty in the treatment of plateau iris, with 87% of eyes remaining open after a single iridoplasty and a mean follow-up time of 79 months.

## Conclusions

In summary, acute angle closure in young patients under age 20, especially those without significant ophthalmic history, should prompt a suspicion of plateau iris syndrome. Reports of plateau iris in this population are rare but should be considered in the differential diagnosis to properly control bilateral glaucoma. Plateau iris is difficult to diagnose without gonioscopy, where the “double hump” sign is classically observed. Ultrasound biomicroscopy may be used to confirm the diagnosis. Diagnosis of plateau iris may warrant peripheral laser iridoplasty and subsequent regular monitoring.
